# Short-Term Nutritional Support for Infants With Unrestricted Ventricular Septal Defects to Promote Postoperative Recovery

**DOI:** 10.3389/fped.2022.888375

**Published:** 2022-05-19

**Authors:** Qi-Liang Zhang, Shi-Hao Lin, Wen-Hao Lin, Hua Cao, Qiang Chen

**Affiliations:** ^1^Department of Cardiac Surgery, Fujian Branch of Shanghai Children's Medical Center, Fuzhou, China; ^2^Fujian Children's Hospital, Fuzhou, China; ^3^Department of Cardiac Surgery, College of Clinical Medicine for Obstetrics & Gynecology and Pediatrics, Fujian Medical University, Fuzhou, China; ^4^Fujian Key Laboratory of Women and Children's Critical Diseases Research, Fujian Maternity and Child Health Hospital, Fuzhou, China

**Keywords:** preoperative nutritional support, unrestricted ventricular septal defect, nutritional status, infants, postoperative recovery

## Abstract

**Objective:**

This study is aimed to explore the effect of short-term nutritional support for infants with unrestricted ventricular septal defects on improving preoperative nutritional status and promoting postoperative recovery.

**Methods:**

The clinical data of 35 infants with unrestricted ventricular septal defects who were treated with 2 weeks of nutritional support in our hospital from December 2020 to March 2021 were analyzed retrospectively. The clinical data of 38 infants with unrestricted ventricular septal defects who were treated in our hospital from May 2020 to October 2020 were selected as controls.

**Results:**

The preoperative body weight, preoperative albumin, preoperative prealbumin, and preoperative hemoglobin in the intervention group were significantly higher than those in the control group (*P* < 0.05). The postoperative ventilator time, intensive care time, and discharge time in the intervention group were significantly shorter than those in the control group (*P* < 0.05).

**Conclusion:**

Performing 2 weeks of nutritional support for infants with unrestricted ventricular septal defects can improve their preoperative nutritional status and promote postoperative recovery.

## Introduction

Malnutrition is a common and serious complication that has been proven to negatively impact the growth and neurocognitive development of children (1). Feeding difficulty, improper feeding, insufficient calorie intake, and increased energy demand are important causes of malnutrition in children with congenital heart disease (2, 3). Studies have shown that most children with congenital heart disease suffer from malnutrition, and malnutrition is considered to be the main cause of morbidity and death in children with congenital heart disease, especially in the perioperative period (4, 5).

Congenital heart disease surgery has been shown to have a positive correlation with weight gain. However, poor nutritional status itself can significantly worsen postoperative nutritional status, affect postoperative recovery, and lead to increased complications, delayed hospital stay, and increased mortality (6). Therefore, it is very important to adjust the nutritional status of children during the perioperative period, especially those with limited nutritional reserves.

Ventricular septal defects are the most common congenital heart diseases, and unrestricted ventricular septal defects are severe. Due to the presence of several left-to-right shunts, pulmonary hypertension, even congestive heart failure, will occur in the early stage. Children need surgical treatment in infancy, and malnutrition is a common problem before surgery (7). To improve the preoperative nutritional status of infants with unrestricted ventricular septal defects and to promote postoperative recovery, we provided short-term nutritional support before surgery.

## Methods

This study was approved by the ethics committee of our hospital and strictly adhered to the tenets of the Declaration of Helsinki. This study included human subjects, and it was retrospective and only data were extracted. In addition, all patients' guardians signed an informed consent form before the study.

**Figure 1:**
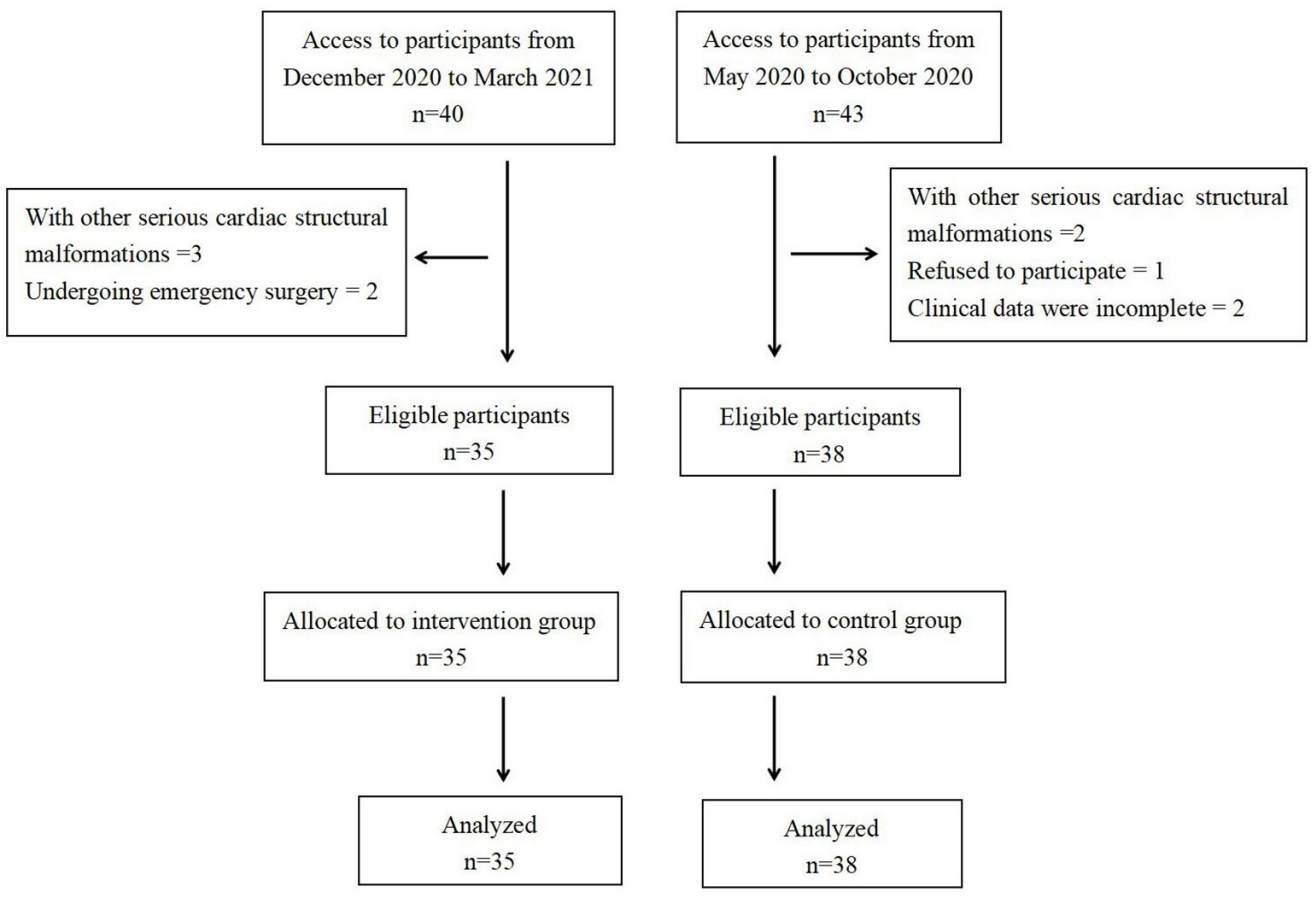
The frame of the study.

In this study, the clinical data of 35 infants with unrestricted ventricular septal defects who were treated with 2 weeks of nutritional support before surgery in our hospital from December 2020 to March 2021 were analyzed retrospectively. The clinical data of 38 infants with unrestricted ventricular septal defects who were not provided preoperative nutritional support in our hospital from May 2020 to October 2020 were selected as the control group (Figure 1). All operations were performed by the same surgical team. We compared the clinical data of the two groups to evaluate the effect of short-term nutritional support before surgery on improving preoperative nutritional status and promoting postoperative recovery in infants with unrestricted ventricular septal defects.

Inclusion criteria were as follows: (1) infants with an unrestricted ventricular septal defect and (2) the patient's condition was relatively stable, and operative treatment was performed within 1–2 months. Exclusion criteria were as follows: (1) infants with liver failure, kidney failure; (2) infants with other serious cardiac structural malformations; (3) infants who underwent emergency surgery during the process of nutritional support; (4) clinical data were incomplete; and (5) infants of parents who refused to participate in this study.

The detailed demographic data were collected, such as gestational weeks, birth weight, weight-for-age *z* scores at birth, head circumference at birth, size of ventricular septal defect, types of ventricular septal defects, pulmonary artery pressure, and preoperative comparisons, such as trisomy 21, renal insufficiency, liver insufficiency, patients needing respiratory support at pre-surgery, respiratory tract infection, use of diuretics, and use of dopamine at pre-surgery. Preoperative data included age at operation, preoperative body weight, preoperative weight-for-age *z* scores, preoperative head circumference, weight gain, change in weight-for-age *z* scores, preoperative albumin, preoperative prealbumin, and preoperative hemoglobin. Operation data included operation time, cardiopulmonary bypass time, and aortic clamping time. Postoperative data included ventilator time, intensive care time, discharge time, and complications. Weight and head circumference were routinely measured at admission and 1 day before surgery in our hospital. The patients in the control group underwent surgery after completing the examination. The time between admission and surgery was very short. The preoperative examination included albumin, prealbumin, and hemoglobin. The intervention group received nutritional support therapy for 2 weeks after admission. The albumin, prealbumin, and hemoglobin were examined on admission and 1 day before surgery. In this study, preoperative weight and head circumference of the two groups were measured 1 day before surgery. The albumin, prealbumin, and hemoglobin in the control group were included in the preoperative examination. The albumin, prealbumin, and hemoglobin of the intervention group were examined 1 day before surgery. Weight gain and change in weight for age *z* scores refer to the changes from birth to 1 day before surgery.

Liver insufficiency was defined as Alanine Aminotransferase (ALT) that was >2 times the normal value of the same age, or total bilirubin that was >40 mg/L. The normal ALT value in our hospital is 8–71 U/L. Renal insufficiency was defined as two times the upper limit of the normal value of serum creatinine of the same age or two times the basic value of serum creatinine. The normal value of serum creatinine in our hospital is 13–33 U/L. Pulmonary artery pressure measurement method: the pulmonary artery pressure was estimated based on tricuspid regurgitant velocity. The differential pressure between the right atrium and right ventricle was calculated by the simplified Bernoulli equation (Δ*P* = 4 × flow rate^2^). The pulmonary artery pressure is estimated by the differential pressure between the right atrium and right ventricle plus the right atrial pressure.

While providing nutritional support, severe heart failure with the proclivity to systemic hypoperfusion was occurred, and the patients needed mechanical ventilation. These patients needed emergency surgery and were not suitable for continuing nutritional support. In this study, two patients in the intervention group underwent emergency surgery during nutritional support.

### Nutritional Support Methods

The infants in the intervention group were admitted to the hospital for nutritional support treatment that 2 weeks before the operation was expected to occur. We set up a nutritional support team to evaluate the baby's nutritional status and nutritional intake every day and to develop a nutritional intake plan for the day. We used the Schofield equation to determine the caloric requirements. According to the guidelines outlined by Leonberg et al., a target caloric intake was 120–150 kcal/kg/day (600 kJ/kg/day), which can ensure adequate energy intake and eliminate the risk of refeeding syndrome. After admission, all infants were fed with bottles, with a target feeding dose of 120–150 kcal/kg/day (600 kJ/kg/day). If the target calorie cannot be achieved with breast milk or regular formula, a breast milk fortifier or high-calorie milk powder can be added. If the child has feeding difficulties and cannot be fed by mouth, nasal feeding can be used. Nasal feeding was performed once every 3 h, with a push pump at a constant speed. The initial feeding session was completed in 20 min. If the infants vomited, then the infusion speed was decreased to 30 or 40 min until vomiting no longer occurred. If the goal cannot be achieved through enteral nutrition alone, supplemental Parenteral nutrition (PN) should be added to meet the target fluid and energy requirements. The proteins in PN were started at 1–1.5 g/kg/day and then 1–1.5 g/kg/day was added every day. Carbohydrates were started at 4–6 mg/kg/min and then 2 mg/kg/min was added every day, and the goal was 12–14 mg/kg/min (45% of calories). Lipids were started at 1 g/kg/day and then 0.5–1 g/kg/day was added every day, and the goal was 3 g/kg/day (40% of calories) (8). When the feeding intolerance symptoms, such as abdominal distention, diarrhea, and constipation appeared, we adopted treatment of reducing the amount of feeding, increasing PN, adding probiotics, and using glycerin enema. In the intervention group, there were four patients with feeding intolerance, the symptoms were relieved after the above symptomatic treatment, and no necrotizing enterocolitis complications occurred.

The infants in the control group did not receive nutritional support, so the process and method of feeding were led by their parents. All the infants in the control group were fed on demand without parenteral nutrition support. Among them, 12 patients were exclusively breastfed, eight were formula fed, and 18 were mixed fed. Four patients who were exclusively breastfed received an addition of breast milk fortifier, and six patients who were mixed fed or formula fed received an addition of high calorie formula. Infants in the intervention group were fed on demand before the nutritional intervention, and the process and method of feeding were also led by their parents. Among them, nine patients were exclusively breastfed, six were formula fed, and 20 were mixed fed. Two patients who were exclusively breastfed received an addition of breast milk fortifier, and five patients who were mixed fed or formula fed received an addition of high calorie formula.

### Statistical Analysis

SPSS 25.0 software was used for statistical analysis. The quantitative data were expressed as the mean ± standard deviation (SD), the continuous data were in accordance with normal distribution by normal distribution test, *t*-test was used for statistical analysis, and the chi-square test was used to compare the qualitative data between the groups. The difference was statistically significant (*P* < 0.05).

## Results

There was no significant difference in the demographic data, such as gestational weeks, birth weight, weight-for-age *z* scores at birth, head circumference at birth, weight at admission, weight for age *z*-scores at admission, head circumference at admission, size of ventricular septal defect, types of ventricular septal defects, pulmonary artery pressure, and preoperative comparisons, such as trisomy 21, renal insufficiency, liver insufficiency, patients needing respiratory support, respiratory tract infection, use of diuretics, and use of dopamine, between the two groups (*P* > 0.05; Table 1).

**Table 1:** Comparison of the demographic data between the two groups [mean ± SD or (%)].

	**Intervention group**	**Control group**	***P*-Value**
Number	35	38	
Gestational age (week)	38.8 ± 1.3	38.5 ± 1.1	0.646
Birth weight (kg)	3.3 ± 0.3	3.4 ± 0.4	0.472
Weight for age *z*-scores at birth	0.26 ± 0.08	0.48 ± 0.12	0.289
Birth head circumference (cm)	32.4 ± 0.9	32.2 ± 1.0	0.728
Weight at admission (kg)	4.5 ± 0.6	4.4 ± 0.5	0.415
Weight for age *z*-scores at admission	−1.74 ± 0.65	−1.85 ± 0.76	0.383
Head circumference at admission (cm)	34.8 ± 0.9	34.6 ± 0.7	0.569
Size of ventricular septal defect (cm)	7.5 ± 0.8	7.8 ± 0.9	0.492
**Types of ventricular septal defect type**
Perimembranous ventricular septal defect	29 (82.9%)	30 (78.9%)	0.672
Subpulmonic ventricular septal defect	6 (17.1%)	8 (21.1%)	
Pulmonary artery pressure (mmHg)	58.0 ± 8.8	58.4 ± 9.6	0.551
Duration of admission prior to surgery (day)	15.2 ± 0.6	3.4 ± 1.3	0.000
Age of operation (day)	54.3 ± 12.5	52.2 ± 12.7	0.682
Trisomy 21	0	0	-
Preoperative renal insufficiency	0	0	-
Preoperative liver insufficiency	1 (2.9%)	1 (2.6%)	0.953
Preoperative respiratory tract infection	5 (14.3%)	6 (15.8%)	0.858
Preoperative patients needing respiratory support at pre-surgery	7 (20%)	9 (23.7%)	0.704
Patients needing diuretics at pre-surgery	8 (22.9%)	9 (23.7%)	0.933
Patients needing dopamine at pre-surgery	6 (26.1%)	6 (15.8%)	0.876

Compared with the preoperative nutritional data of the two groups, body weight, weight-for-age *z* scores, weight gain, change in weight-for-age *z* scores, albumin, prealbumin, and hemoglobin in the intervention group were significantly higher than those in the control group (*P* < 0.05). There was no significant difference in operation time, cardiopulmonary bypass time, or aortic clamping time between the two groups (*P* > 0.05). The postoperative ventilator time, intensive care time, and discharge time in the intervention group were significantly shorter than those in the control group (*P* < 0.05). Albumin, prealbumin, and hemoglobin of the intervention group increased after short-term nutritional support therapy (Table 2).

**Table 2:** Comparison of the perioperative data between the two groups [mean ± SD or (%)].

	**Intervention**	**Control**	***P*-Value**
	**group**	**group**	
Number	35	38	
Preoperative body weight (kg)	5.0 ± 0.8	4.4 ± 0.5	0.009
Preoperative weight for age *z*-scores	−0.81 ± 0.22	−1.85 ± 0.76	0.005
Preoperative head circumference (cm)	35.3 ± 1.1	34.6 ± 0.7	0.189
Weight gain (kg)	1.7 ± 0.5	1.1 ± 0.3	0.023
Change in weight for age *z* scores	−1.08 ± 0.26	−2.31 ± 0.84	0.012
Preoperative albumin (g/L)	35.8 ± 2.8	31.4 ± 1.9	0.033
Preoperative prealbumin (g/L)	209.2 ± 19.8	176.1 ± 15.1	0.032
Preoperative hemoglobin (g/L)	113.1 ± 10.2	104.8 ± 13.3	0.035
Change in albumin (g/L)	3.2 ± 1.3	–	–
Change in prealbumin (g/L)	22.4 ± 3.6	–	–
Change in hemoglobin (g/L)	9.8 ± 2.2	–	–
Operation time (h)	3.1 ± 0.3	3.1 ± 0.2	0.177
Cardiopulmonary bypass time (h)	1.5 ± 0.2	1.6 ± 0.3	0.712
Aortic cross-clamping time (min)	36.8 ± 4.5	35.9 ± 4.7	0.833
Mechanical ventilation time (day)	3.5 ± 0.7	4.2 ± 1.0	0.017
Intensive care time (day)	4.8 ± 0.5	5.9 ± 0.8	0.022
Discharge time (day)	12.5 ± 2.7	15.1 ± 3.9	0.029

Compared with the postoperative complications, the incidence of postoperative pulmonary infection in the intervention group was significantly lower than that in the control group (*P* < 0.05). There was no significant difference in complications, such as liver insufficiency, renal insufficiency, poor incision healing, or gastrointestinal bleeding, between the two groups. No complications, such as death, heart failure, or malignant arrhythmia, occurred in the two groups (Table 3).

**Table 3:** Comparison of the postoperative complications between the two groups [mean ± SD or (%)].

	**Intervention group**	**Control group**	***P*-Value**
Number	35	38	
Pulmonary infection	4 (11.4%)	11 (28.9%)	0.048
Heart failure	0	0	–
Malignant arrhythmias	0	0	–
Liver insufficiency	2 (5.7%)	2 (5.3%)	0.933
Renal insufficiency	1 (2.9%)	2 (5.3%)	0.605
Surgical wound bad healing	0	1	–
Gastrointestinal bleeding	1	0	–
Death	0	0	–

## Discussion

Congenital heart disease is the most common congenital structural malformation, which accounts for approximately 28% of all congenital malformations and is the main cause of death in children ages 0–5 years. Surgery is the main method of treatment for patients with congenital heart disease. This type of operation is often performed for patients in cardiac arrest, which causes considerable trauma to the body and increases nutrition consumption. It is necessary to have a good nutritional level to improve the patient's tolerance to the operation. Therefore, the preoperative nutritional status of children is an important factor that contributes to the effect of the operation on postoperative rehabilitation (9, 10). However, due to cardiac function disturbance and to the decline or disorder of cardiac pumping function in children with congenital heart disease, a disease with an earlier onset indicates that the condition is more serious, which suggests that patients are more difficult to feed and experience difficulty in ingesting food. At the same time, a decline in cardiac function leads to cardiac insufficiency, circulatory blood disorders, gastrointestinal digestion and absorption disorders, malnutrition, and hypoproteinemia. Several studies have shown that malnutrition is more common in hospitalized children with congenital heart disease, especially those with high-risk factors, such as heart failure and pulmonary hypertension (11–13). Therefore, providing reasonable nutritional support for children with congenital heart disease before surgery and improving the nutritional status before surgery can effectively improve the effect of surgery, promote recovery after surgery, and improve the clinical outcome after surgery (14).

An unrestricted ventricular septal defect is a common congenital heart defect. Due to the large ventricular septal defect and a large number of left-to-right shunts, congestive heart failure and severe pulmonary hypertension easily develop in patients in the early stage; it is difficult to feed these patients and difficult for them to ingest food. Therefore, malnutrition is often an obvious occurrence before surgery. Because of the obvious symptoms, patients usually need to receive surgical treatment as a newborn or in infancy. The nutritional status before the operation obviously affects the patient's tolerance to the operation and the patient's speed of recovery after the operation. In order to explore methods to improve the nutritional status of infants with unrestrictive ventricular septal defect before surgery, our department carried out an institutional change in nutritional practices in December 2020. We began to implement short-term nutritional support for 2 weeks. During this period, we performed nutritional support therapy for 2 weeks for all infants with unrestrictive ventricular septal defect who underwent confine operation. Nutritional support therapy was carried out as described in the article above. After 4 months, we have implemented the short-term nutritional support for 35 infants. In order to a preliminary assessment of the effect of short-term nutritional support, we selected 38 infants without nutritional support from May 2020 to October 2020 as a comparison.

Nutritional status is a potentially modifiable risk factor, and many studies have shown that optimizing preoperative nutritional status in children with congenital heart disease can improve short-term and long-term outcomes (15). Gongwer et al. (10) observed that compared with the control group, the WAZ of the intervention group obviously increased after a standardized nutrition plan. In addition, El-Koofy et al. used a nutrition rehabilitation program for 3 months in 50 infants with left-to-right cardiac shunts. They recorded that after the nutrition rehabilitation program, the incidence of moderate malnutrition was decreased from 14 to 6%, and the incidence of severe malnutrition was decreased from 20 to 4% (16). In addition, Mehta et al. (17) described that if children with heart disease did not receive any nutritional intervention before planned heart surgery, the Weight-for-age z score (WAZ) and Weight-for-height z score (WHZ) of half of the infants indicated that they were severely underweight at the time of surgery. This study also reached a similar conclusion, which found that in comparison with the control group, the intervention group had higher preoperative body weight, albumin, prealbumin, and hemoglobin and had better preoperative nutritional status. This shows that short-term nutritional support should be provided for 2 weeks before the operation. Setting target calories, using a breast milk fortifier, high calorie milk powder, or even parenteral nutrition, and strictly managing the energy intake of children can effectively improve the nutritional status of unrestricted ventricular septal defects. Due to the Gut hypoperfusion caused by moderate-to-severe heart failure of unrestricted ventricular septal defects, feeding intolerance and necrotizing enterocolitis are the complications that we cannot ignore. Feeding intolerance symptoms, such as abdominal distention, diarrhea, and constipation, need to be closely observed during the feeding process. Once they appear, we should take timely measures, such as reducing the amount of feeding, increasing PN, adding probiotics, and using glycerin enema. In the intervention group, there were four patients with feeding intolerance, the symptoms were relieved after the symptomatic treatment, and no necrotizing enterocolitis complications occurred.

It is well known that protein accumulation and energy deficiency in critically ill children significantly affect the recovery of children and the occurrence of complications, which are risk factors for prolonged mechanical ventilation and intensive care unit (ICU) hospitalization (18). Kelleher et al. (19) reported that postoperative WAZ was negatively correlated with mechanical ventilation time, the length of hospital and ICU stay. In addition, Lim et al. reported that preoperative WAZ and HAZ < –2 significantly increased the duration of mechanical ventilation, the duration of intensive care, and the duration of hospitalization after the operation (15). This study also showed that short-term preoperative nutritional support can significantly improve the preoperative nutritional status of infants with unrestricted ventricular septal defects and can significantly shorten the duration of mechanical ventilation, intensive care, and hospital stay after surgery and promote the recovery of children after operation.

This study still has some limitations. First of all, this study was a single-center retrospective study, and the data on how much did the hemoglobin and prealbumin increase and the cost of nutritional support cannot be obtained. This is a comparison of patients from two different periods so that there was unplanned than the fact that there were confounders related to medical decision-making that cannot be identified. All of these limited the objectivity of the study. Second, due to the disadvantage of the retrospective study, the time points of some indexes in the two groups could not be completely consistent, such as preoperative albumin, preoperative hemoglobin, and preoperative prealbumin. Some indexes could not be achieved, for example, the control group could not provide the changes of 2 weeks prior to surgery. In the next prospective study, we will solve these problems. Third, the sample size of this study was small. Fourth, this study only explored the typical cases of unrestricted ventricular septal defects in children with left-to-right shunts and did not study the effect of nutritional support on other congenital heart diseases. In the future, it is necessary to conduct a multicenter prospective study to objectively evaluate the effect of short-term preoperative nutritional support on improving the postoperative prognosis of patients with congenital heart disease.

## Conclusion

Performing 2 weeks of nutritional support for infants with unrestricted ventricular septal defects can improve their preoperative nutritional status and promote postoperative recovery.

## Data Availability Statement

The original contributions presented in the study are included in the article/supplementary material, further inquiries can be directed to the corresponding author.

## Ethics Statement

The studies involving human participants were reviewed and approved by Ethics Committee of Fujian Children's Hospital. Written informed consent to participate in this study was provided by the participants' legal guardian/next of kin.

## Author Contributions

Q-LZ designed the study, acquired and interpreted the data, and drafted the manuscript. S-HL analyzed and interpreted the data. W-HL acquired and analyzed the data. HC has made substantial contributions to conception and design. QC was involved in the analysis and interpretation of data, revising the manuscript, and gave final approval of the version to be published. All authors contributed to the article and approved the submitted version.

## Conflict of Interest

The authors declare that the research was conducted in the absence of any commercial or financial relationships that could be construed as a potential conflict of interest.

## Publisher's Note

All claims expressed in this article are solely those of the authors and do not necessarily represent those of their affiliated organizations, or those of the publisher, the editors and the reviewers. Any product that may be evaluated in this article, or claim that may be made by its manufacturer, is not guaranteed or endorsed by the publisher.
